# Editorial: Systems modeling: Approaches and applications–Volume II

**DOI:** 10.3389/fmolb.2022.1048727

**Published:** 2022-10-21

**Authors:** Daniel Garrido, Alberto J. M. Martín, Ernesto Pérez-Rueda

**Affiliations:** ^1^ Department of Chemical and Bioprocess Engineering, School of Engineering, Pontificia Universidad Católica de Chile, Santiago, Chile; ^2^ Centro Científico y Tecnológico de Excelencia Ciencia & Vida, Fundación Ciencia & Vida, Escuela de Ingeniería, Facultad de Ingeniería, Arquitectura y Diseño, Universidad San Sebastián, Santiago, Chile; ^3^ Instituto de Investigaciones en Matemáticas Aplicadas y en Sistemas, Universidad Nacional Autónoma de México, Unidad Académica Yucatán, Mérida, Mexico

**Keywords:** systems modeling, biomedical, microbial biochemistry, metabolic engineering, theoretical approaches

## Introduction

The development of modeling tools has permitted an increased understanding of how components in different systems interact and behave. Thus, systems modeling has led to critical advances in several areas, such as medicine, biotechnology, and engineering. Applications include the study of ecological models, diseases and the impact of treatments, microorganism responses to specific environments, and the interactions between biomolecules.

The main goal of this Research Topic (*Systems Modeling: Approaches and Applications–Volume II*) was to bring together novel biological applications and studies on systems modeling. We were thrilled to witness the great interest in the field and the high number of manuscripts submitted. Broadly, works published in this section could be classified in four main categories: biomedicine, metabolic engineering, microbial biochemistry, and theoretical approaches and novel applications.

Among the biomedical articles of the number, Ponce-de-Leon et al., expanded a hybrid multi-scale model including time and space variables of fibroblast spheroids, which could be useful for addressing cancer cell resistance by including time, geometric and population variability. Prybutok et al. extended an agent-based modeling framework to design alternatives in Chimeric antigen receptor (CAR) T-cell therapy. This approach might accelerate the discovery of novel strategies against solid tumors. Later, Ordaz-Arias et al. presented a regulatory network of macrophages, in order to study their plasticity, adaptability, and heterogeneity, and finding oscillations derived from the network structure. Zinovyev et al. developed a model of the cell cycle at the single cell level including internal dynamical cycles and switches. It predicted with great accuracy cell doubling times. Lecca and Ihekwaba-Ndibe et al. focused on DNA repair mechanisms, developing a mathematical model of the gene regulatory network of this biological process. Applications of the model include evaluation of the effect of certain mutations and control of participating genes. Gupta et al. studied the molecular mechanisms involved in the response of macaques to malaria using transcriptomics and metabolic modeling. Gupta et al. focused on pathogen detection pathways and inflammasome assembly, developing a comparative analysis identifying points of control for maintaining immune balance. Finally, Gil et al. developed a 3D model of calcium signaling pathways in T-cells to investigate how calcium microdomains occurred and included the role of ryanodine receptors in TCR/CD3 stimulation.

Microbial biochemistry articles include a study of Spolaor et al., who developed a mathematical model of the effect of hypotonic shock on calcium homeostasis and signaling pathways in *Saccharomyces cerevisiae*. The model included mechanosensitive channels, and provided an interpretation of regulatory processes in wild type and mutant yeasts. Verhagen et al. addressed resource limitations on optimal proteome allocations developing a resource-dependent kinetic model of *S. cerevisiae*. The model predicted proteome adaptations to multiple conditions with changing resources, and could be useful for industrial yeast applications. Rajeshkannan et al. presented a mathematical model of the GAL regulon in *S. cerevisiae*, which showed that binding affinities between regulatory proteins modulate gene expression at the single cell and population levels. Finally, Posada-Reyes et al. analyzed polymorphic interactions in the genomes and pangenome of *Mycobacterium tuberculosis*. They presented an epistatic network for this microorganism and identified targets of co-selection, contributing to our understanding of *M. tuberculosis* pathogenesis.

Metabolic engineering manuscripts included Landon et al., developing an analysis pipeline to interpret metabolic reaction fluxes, integrating machine learning, dimensionality reduction and network analysis. The work presented by Landon et al. focused on the *Mycoplasma genitalium* whole-cell model and the contribution of the model to understand gene knock-outs in a minimal genome. Doan et al. presented a coarse-grained mathematical model, comprising a micromolecular and a macromolecular component, aimed to represent a cell proteome during microbial growth in a bioprocess. Köbis et al. developed a constraint-based model that considers a time-optimal control problem, which allows to determine the fastest possible adaptation of a system to a cellular state. Boada et al. used multiobjective optimization for tuning gene circuits composed of a controller and a biosensor controlling metabolic pathways. This study might contribute to optimizing microbial cell processes and system robustness and stability. Finally, Lazaro et al. constructed a mathematical model that integrates two major steps in bioprocessing: single cell growth captured by a genome-scale metabolic model with bioreactor dynamics. This work used production of citralamate in *Escherichia coli* as case study, and might have important applications in biotechnological processes.

At last, other articles presented theoretical approaches, code and novel applications. Stoll et al. introduced a framework modeling dynamic population of interacting cells, based on probabilistic simulations and using TNF-induced cell death as case study. Litwin et al. addresses the task of determining model parameters in an ODE-based model, proposing a 2D likelihood approach to aid in optimal experimental design for parameter determination. Selvaggio et al. addressed the need of quantitative data required for calibrating model parameters. They proposed a hybrid model integrating ODE and logical formalities to describe biological complexity in layers and their communication. Medina-Ortiz et al. maximized the performance of predictive models in protein engineering, generalizing property-based encoders. This work contributes to predictive protein engineering without increasing model complexity. Massing et al. reviews generalized modeling, an approach to conventional dynamic modeling, highlighting recent advances and providing an application guide for this approach. Voit and Olivença focused on the Biochemical Systems Theory, an ODE-based approach for biochemical reaction analysis and simulation, expanding this theory to include stochasticity, discreteness and addressing time delays.

We consider that the field of systems modeling is in expansion, supported by the great quality and number of manuscripts included in this special issue. We appreciate the great interest and reception of the community, and hope that this special number is of interest for researchers in the field of computational biology, biochemistry, biomedicine, bioengineering and mathematics.

